# One in five patients with Duchenne muscular dystrophy dies from other causes than cardiac or respiratory failure

**DOI:** 10.1007/s10654-021-00819-4

**Published:** 2021-11-21

**Authors:** Lisa Wahlgren, Anna-Karin Kroksmark, Mar Tulinius, Kalliopi Sofou

**Affiliations:** 1 The Queen Silvia Children’s Hospital, Sahlgrenska University Hospital, Gothenburg, Sweden; 2 Department of Pediatrics, Institute of Clinical Sciences, Sahlgrenska Academy, University of Gothenburg, Gothenburg, Sweden; 3 Department for Health and Rehabilitation/Physiotherapy, University of Gothenburg, Gothenburg, Sweden

**Keywords:** Duchenne muscular dystrophy, Epidemiology, Prevalence, Survival, Neuromuscular, Cause of death

## Abstract

Duchenne muscular dystrophy (DMD) is a severe neuromuscular disorder with increasing life expectancy from late teens to over 30 years of age. The aim of this nationwide study was to explore the prevalence, life expectancy and leading causes of death in patients with DMD in Sweden. Patients with DMD were identified through the National Quality Registry for Neuromuscular Diseases in Sweden, the Swedish Registry of Respiratory Failure, pathology laboratories, neurology and respiratory clinics, and the national network for neuromuscular diseases. Age and cause of death were retrieved from the Cause of Death Registry and cross-checked with medical records. 373 DMD patients born 1970–2019 were identified, of whom 129 patients deceased during the study period. Point prevalence of adult patients with DMD on December 31st 2019 was 3.2 per 100,000 adult males. Birth prevalence was 19.2 per 100,000 male births. Median survival was 29.9 years, the leading cause of death being cardiopulmonary in 79.9% of patients. Non-cardiopulmonary causes of death (20.1% of patients) mainly pertained to injury-related pulmonary embolism (1.3 per 1000 person-years), gastrointestinal complications (1.0 per 1000 person-years), stroke (0.6 per 1000 person-years) and unnatural deaths (1.6 per 1000 person-years). Death from non-cardiopulmonary causes occurred at younger ages (mean 21.0 years, SD 8.2; *p* = 0.004). Age at loss of independent ambulation did not have significant impact on overall survival (*p* = 0.26). We found that non-cardiopulmonary causes contribute to higher mortality among younger patients with DMD. We present novel epidemiological data on the increasing population of adult patients with DMD.

## Introduction

Duchenne muscular dystrophy (DMD) is a rare neuromuscular disease with an estimated global birth prevalence of 19.8 per 100,000 live male births [1]. In western Sweden, the estimated prevalence is 21.5 per 100,000 school-aged boys [2]. DMD is caused by mutations in the dystrophin gene (Xp21.2) leading to total absence or nearly undetectable truncated levels of the dystrophin protein in skeletal muscle [3, 4]. Lack of dystrophin causes the muscle fibers to degenerate and be replaced with fat and connective tissue resulting in a progressive loss of muscle strength [5–7]. DMD leads to loss of ambulation before adolescence and, without treatment, life expectancy does not reach beyond late teens [8].

The two most common causes of death in DMD are respiratory and cardiac failure [9–11]. Chronic respiratory failure is attributed to weakness of the respiratory muscles and reduced chest wall compliance due to fibrotic changes of the chest wall muscles and scoliosis [12]. Weakness in expiratory muscles ultimately results in an increase in residual volume and decreased coughing ability. The lung compliance may also be affected because of micro-atelectatic changes [12]. Acute respiratory failure is common, often triggered by pneumonia or secretion stagnation. The heart is also affected by the lack of dystrophin, beginning with myocardial fibrosis in the left ventricular wall behind the mitral valve, to eventually affect the entire left ventricle [13]. Dilated cardiomyopathy is the most common cardiac involvement in patients with DMD [14], and increases the risk of premature death [15].

Improved care with corticosteroid therapy, respiratory care, prevention and treatment of cardiomyopathy and scoliosis treatment has had a positive impact on both life expectancy and the course of the disease [10, 11, 16–20]. Treatment with corticosteroids has been shown to slow down the disease progression with respect to muscle strength, motor function including ambulation, scoliosis and pulmonary function [16, 21–24]. Timely initiation of non-invasive mechanical ventilation, along with mechanical insufflation-exsufflation and early cardio- protection have been associated with an increasing longevity of a median life span over 30 years [19].

To date, there are limited studies on the evolution of natural history and causes of death in DMD, mainly restricted to small patient populations and specialized neuromuscular clinics [10, 11, 17–19]. The aim of this nationwide study was to explore the prevalence, life expectancy and leading causes of death in patients with DMD in Sweden.

## Materials and methods

### Study population

All male patients born since 1st January 1970 who died by 31st December 2019 and were followed at a medical clinic in Sweden with a confirmed diagnosis of DMD were included in the study. The diagnostic criteria applied were typical clinical phenotype of DMD with elevated serum creatine kinase and either (i) muscle biopsy findings compatible with DMD or (ii) pathogenic DMD variants in the dystrophin gene or (iii) confirmed DMD diagnosis in a maternal relative. A typical clinical phenotype was defined as symptom onset before 5 years of age and loss of ambulation before 13 years of age [25]. Since corticosteroid treatment has shown a 3-year delay in loss of ambulation [24], patients who had received corticosteroids and lost ambulation before 16 years of age were considered eligible for inclusion, if fulfilling the diagnostic criteria mentioned above. Patients were excluded from the study if they had an intermediate form between DMD and Becker muscular dystrophy (BMD) defined as loss of ambulation between 13 and 16 years of age without corticosteroid treatment, or BMD defined as loss of ambulation above 16 years of age, or any other form of muscular dystrophy.

### Sources used to identify the study population

**Fig. 1:**
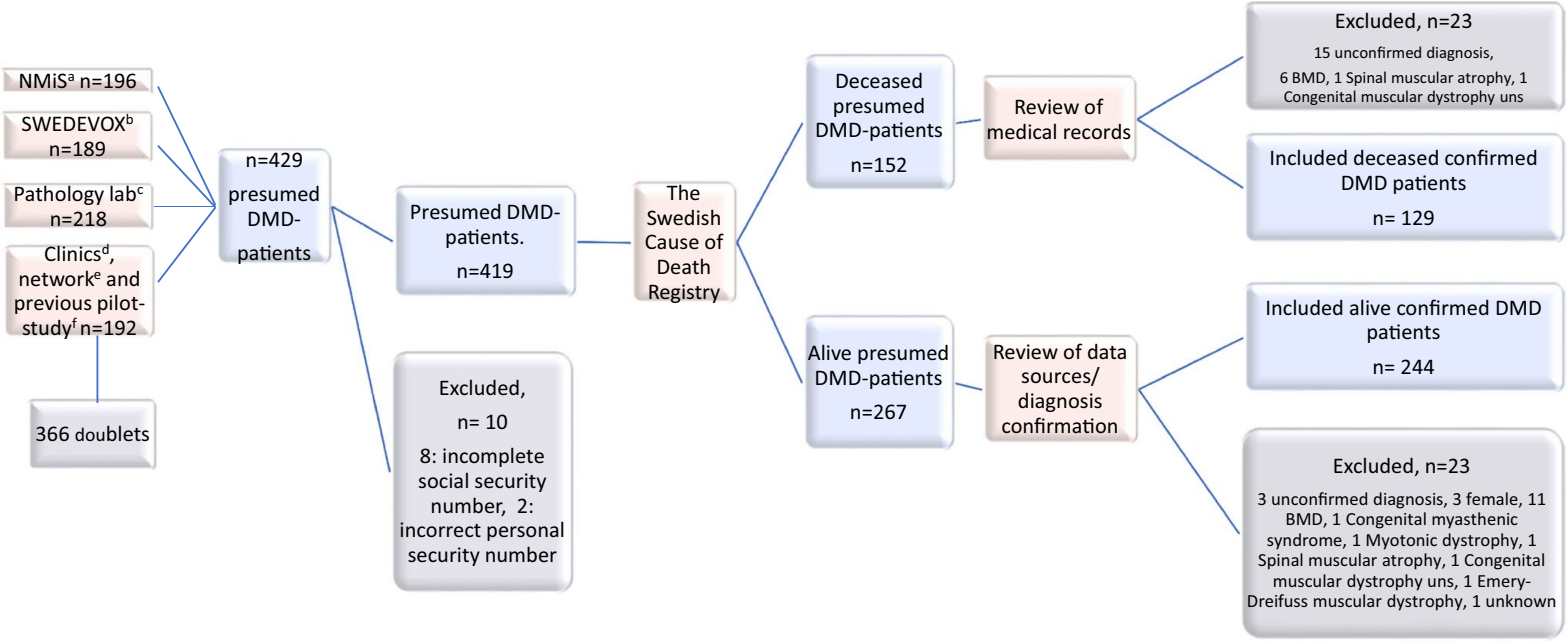
Flowchart: Identification and inclusion procedure of the Swedish patients with DMD born 1970–2019. Patients identified through: **a** the National Quality Registry for Neuromuscular Diseases in Sweden (NMiS.) **b** the Swedish Registry of Respiratory Failure (Swedevox) **c** all pathology laboratories analyzing skeletal muscle biopsies in Sweden d. an inquiry to all neurology clinics and pulmonary clinics in Sweden e. the Swedish neuromuscular network. **f** a pilot study for patients with DMD deceased between 2000 and 2010 [26]. DMD = Duchenne muscular dystrophy, BMD = Becker muscular dystrophy, n = number

Patients were identified via the National Quality Registry for Neuromuscular Diseases in Sweden (NMiS), The Swedish Registry of Respiratory Failure (Swedevox) and all pathology laboratories performing skeletal muscle biopsies in Sweden, i.e. the Sahlgrenska University Hospital in Gothenburg, the Karolinska University Hospital in Stockholm and the University Hospitals in Linköping, Umeå, Uppsala and Lund, which represent the six health care regions in Sweden. In addition, patients were identified through an inquiry to all neurology and respiratory clinics in Sweden, as well as the national network of clinicians working with neuromuscular diseases in Sweden. A pilot study for patients with DMD deceased between 2000–2010 in Sweden was also used [26]. The Cause of Death Registry at the Swedish National Board of Health and Welfare was used to retrieve information on identified patients regarding the date and cause of death (Fig. 1; Flowchart, inclusion process).

### Data collection

Data was collected from the patients’ medical records in a systematic manner with the use of a case report form, which is available upon request. Data included clinical features at onset and during the disease course, age at diagnosis, serum CK at diagnosis, age at loss of ambulation, muscle biopsy findings, genetic findings, family history, corticosteroid treatment as well as age and cause of death. All medical records were reviewed by two pediatric neurologists with expertise in neuromuscular disorders.

#### Data collection for epidemiological analyzes

For patients who were alive by the end of 2019, confirmation of DMD diagnosis was performed for calculation of prevalence and survival analyses. The diagnosis was verified through the NMiS registry and/or through the pathology reports if the latter matched the inclusion criteria, otherwise the medical records were used for confirmation. Data with estimates of the Swedish population were collected from Statistics Sweden, www.scb.se/en. There were 2,711,935 live male births over a period of 50 years (1970–2019). Between 1970–79 there were 537,089 live male births, in 1980–89 there were 515,590, in 1990–99 there were 547,704, in 2000–09 there were 521,728 and in 2010–19 there were 589,824. The Swedish adult male population,18–99 years of age, alive and at risk at the end of 2019 was 4,073,160.

### Leading cause of death

For all deceased patients in Sweden, the cause(s) of death are registered at the Cause of Death Registry at the Swedish National Board of Health and Welfare. To ensure consistency and accuracy in our study, we cross-checked the Cause of Death Registry against patients’ medical records. In the event of discrepancies, we based the leading cause of death on that reported in the medical records. In the event of multiple conditions contributing to death, the most prominent condition leading to death was defined as the leading cause of death. In the event of sudden death at home without any other apparent causes contributing to death (i.e. ventilator malfunction, infection etc.), ‘cardiac arrest’ was reported as the leading cause of death. In the event of accidental or non-accidental injury or poisoning leading to death in connection with the incident this was documented as ‘unnatural cause of death’ (International Classification of Diseases- ICD 10). When the leading cause of death was only based on the Cause of Death Register (i.e. not verified via patient’s medical records), this was documented as ‘non-verified’. For those not verified in the medical records and poorly described in the Cause of Death Registry, the cause of death was documented as ‘unknown’.

### Statistical analysis

Continuous variables are presented with mean and standard deviations (SD) and median and quartile 1 (25%) and quartile 3 (75%) (Q1;Q3). Categorical variables are presented with numbers and percentages. All analyzes were assessed with non-parametric tests. Jonckheere-Terpsta Test was used to analyze the trends over the decades regarding age at diagnosis, age at loss of ambulation (the patients never learning to walk due to other reasons (i.e. cerebral palsy) were excluded in the analyzes) and age at start with corticosteroids. Birth prevalence was calculated as the number of patients diagnosed with DMD during each decade, i.e. 1970–79, 1980–89, 1990–99, 2000–09 and 2010–19 in relation to the total live male births during the same decade. The estimated birth prevalence was divided by the previously estimated prevalence in western Sweden presented by Darin and Tulinius [2]. This is presented as ‘percent of calculated birth prevalence’. Point prevalence for the adult population, born 1970 or later, was calculated as the number of patients with DMD over 18 years of age and known to be alive on December 31st 2019, in relation to the total male population 18–99 years of age in Sweden known to be alive on December 31st 2019. Survival data for the total population, the population divided into decades of birth and the population divided into the six health care regions in Sweden, were described by Kaplan–Meier-curves and analyzed by the Log-Rank test. To ensure that we had a representative population from each study decade we used the ‘percent of calculated birth prevalence’, as defined above. If the percent were under 80% the population of this study decade was considered as non-representative and thus excluded from the survival analyzes. Patients who were alive at the end of the study period were censored. The incidence of non-cardiopulmonary causes of death was calculated as number of cases divided by the study population in person-years. The Fisher´s Non Parametric Permutation Test was used to analyze differences in age between cardiopulmonary causes of death and non-cardiopulmonary. Mean age difference with 95% confidence interval is given. For comparison of non-cardiopulmonary causes of death over the three age-classes < 20, 20–30 and > 30 years of age the Mantel–Haenszel Chi Square test was used. All significance tests were two-sided and conducted at the 5% significance level. Analysis was made using IBM SPSS statistics 25 (© Copyright IBM Corporation 1994, 2021).

### Ethical considerations

The study protocol was approved by the Swedish Ethics Committee (Dnr. 1130–11 and 2019–01,790).

## Results

### Study population

We identified 129 patients with DMD who were born between 1970 and 2019 and deceased during the same time period (Fig. 1; Flowchart, inclusion process). Patient demographics are presented in Table 1, along with population distribution across decades. Diagnosis was based on muscle biopsy findings in 108 (84%), genetic findings in 45 (35%) and family history for DMD in 38 (33%) of the patients. There was no change over time regarding the age at diagnosis (*p* = 0.72) or the age at loss of ambulation (*p* = 0.49). There was a significant decrease in the age at start of corticosteroid treatment between patients born 1970–79 and 1990–99 (*p* = 0.023) and between patients born 1980–89 and 1990–99 (*p* < 0.001).

**Table 1:** Demographics of Swedish deceased DMD patients, born 1970-2019

	Total population	By decade of birth			
	1970–2019 n = 129	1970–1979 n = 26	1980–1989 n = 63	1990–1999 n = 37	2000–2009 n = 3
*Age at diagnosis (n)*	123	25	59	36	3
mean (SD), y	4.83 (1.74)	4.85 (2.08)	4.94 (1.52)	4.50 (1.80)	6.29 (1.95)
Median (Q1;Q3), y	4.61 (3.41;6.09)	4.58 (3.26;6.18)	4.93 (3.90;6.24)	4.36 (3.15;5.87)	7.00 (4.09;7.79)
*Age at LoA (n)*	127	26	61	37	3
mean (SD), y	10.29 (2.08)	10.46 (1.86)	9.91 (2.02)	10.84 (2.32)	10.00 (0.50)
Median (Q1;Q3), y	10.00 (9.00;11.90)	10.65 (9.00;12.00)	10.00 (8.50;11.00)	11.00 (9.20;12.00)	10.00 (9.50;10.50)
*Treated with cc* N (%)	76 (64.4%)	5 (21.7%)	36 (64.3%)	32 (88.9%)	3 (100%)
*Age at start cc (n)*	63	5	29	27	2
mean (SD), y	7.55 (3.95)	14.64 (7.11)	8.44 (2.93)	5.36 (2.03)	6.41 (3.15)
Median (Q1;Q3), y	6.49 (5.06;9.12)	13.48 (12.93;17.09)	7.52 (6.34;10.53)	5.67 (3.92;6.46)	6.41 (4.18;8.64)
*Age at death (n)*	129	26	63	37	3
mean (SD), y	24.38 (7.06)	30.67 (8.22)	24.75 (5.64)	20.12 (4.41)	14.50 (2.03)
Median (Q1;Q3), y	24.34 (19.16;28.56)	30.31 (25.34;37.74)	25.49 (20.72;28.03)	19.85 (17.90;22.09)	14.41 (13.46;15.49)

DMD = Duchenne muscular dystrophy, n = number, SD = standard deviation, y = years Q = quartile, LoA = loss of ambulation, cc = corticosteroids

### Epidemiology

By December 31st 2019, a total of 373 patients born since 1970 with confirmed DMD were identified (Table 2). The DMD population born during the period 1980–2009, i.e. after removing the first and last study decades as non-representative for the entire DMD population, was estimated at 305 patients, representing a birth prevalence of 19.2 per 100,000 live male births. The number of adult patients with DMD in Sweden who were alive on December 31st 2019 was 130 patients, accounting for a point prevalence of 3.2 per 100,000 adult males.

**Table 2:** Epidemiology of Swedish DMD patients (alive and deceased), born 1970–2019

	Total n	Deceased n (%)	Birth prevalence x per 100,000 (% of calculated birth prevalence^a^)
Total population born:			
1970–2019	373	129 (34.6%)	13.8 per 100,000
			(64.2%)
Population divided in birth decades:			
1970–1979	31	26 (83.9%)	5.8 per 100,000
			(27.0%)
1980–1989	99	63 (63.6%)	19.2 per 100,000
			(89.3%)
1990–1999	112	37 (32.7%)	20.5 per 100,000
			(95.3%)
2000–2009	94	3 (3.2%)	18.0 per 100,000
			(83,7%)
2010–2019	37	0 (0.0%)	6.3 per 100,000
			(29.3%)
Population used for survival analyses:			
1980–2009	305	103 (33.8%)	19.2 per 100,000
			(89.3%)

^a^based on the study by Darin and Tulinius [2], with 21.5 per 100,000 school-aged boys in the western Sweden

DMD = Duchenne muscular dystrophy, n = number

### Life span

**Fig. 2:**
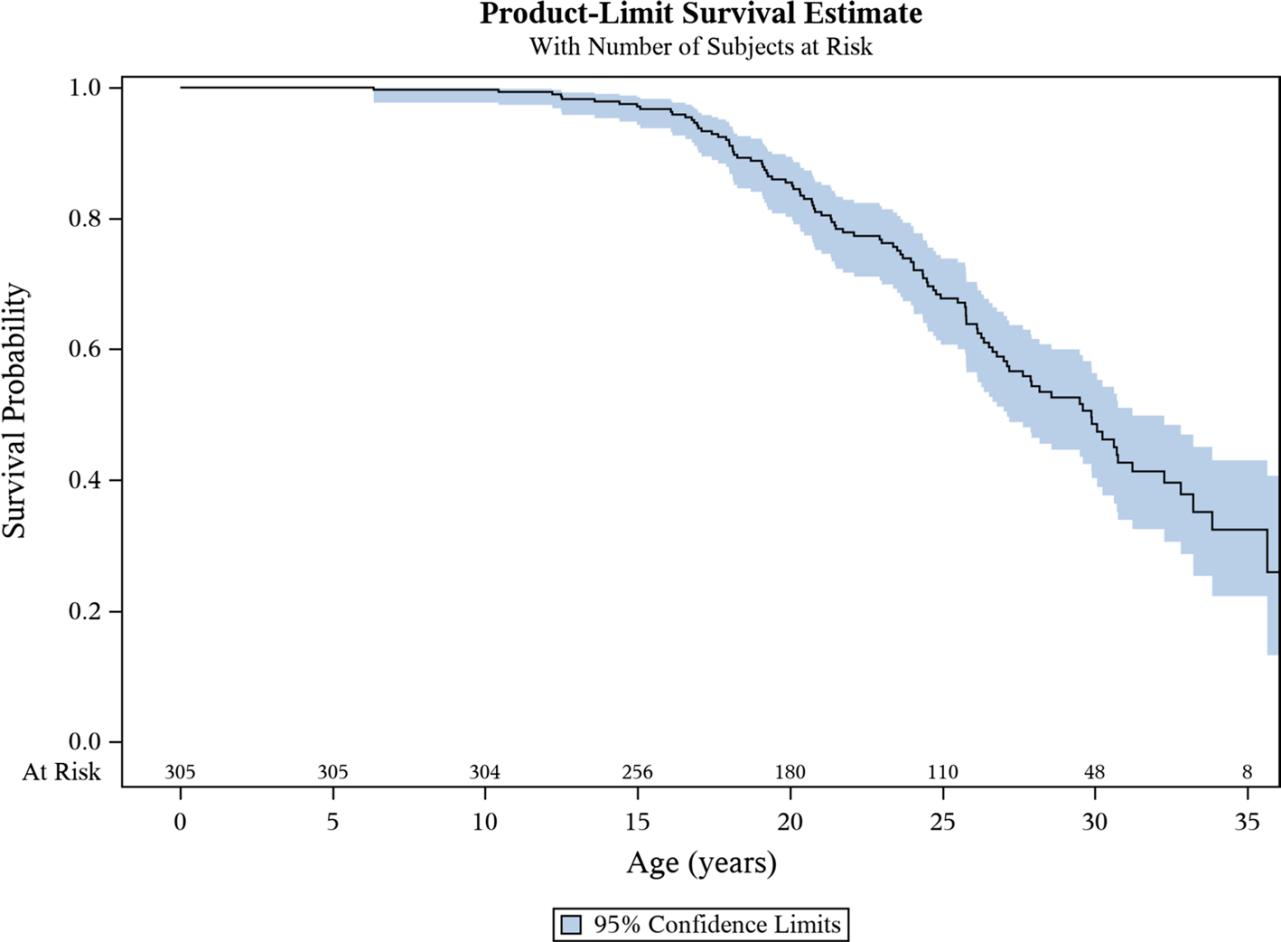
Kaplan–Meier curve for survival estimate for Swedish patients with DMD, born 1980–2009. (including alive patients censored on 31st December 2019). Median survival 29.9 years of age (95% CI 27.1–31.2). Shaded blue area indicates the 95% confidence interval. DMD = Duchenne muscular dystrophy, CI = Confidence Interval

**Fig. 3:**
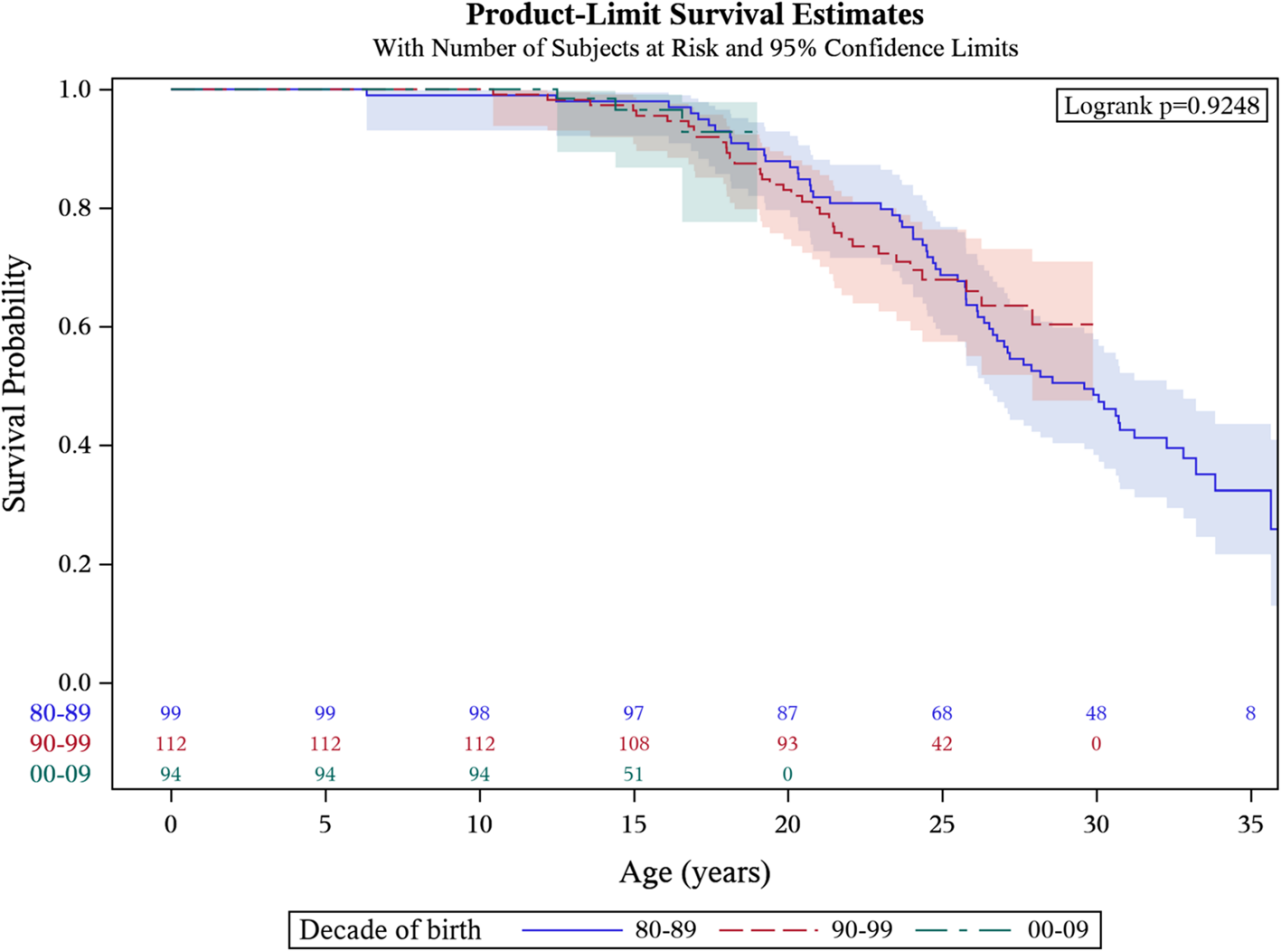
Kaplan–Meier curve for survival estimate for Swedish patients with DMD presented per birth decades for population born 1980–2009. (including alive patients censored on 31st December 2019). Shaded areas indicate the 95% confidence interval. Median survival for patients born 1980–89 was 29.6 years of age (95% CI 26.5–32.3). DMD = Duchenne muscular dystrophy, CI = Confidence Interval

The median survival was 29.9 years of age (95% CI 27.1–31.2) with 75% of patients surviving to 23.6 years of age and 25% of patients surviving to 36.5 years of age (Fig. 2; population born 1980–2009). For patients born 1980–89, the median survival was 29.6 years (95% CI 26.5–32.3) with 75% of patients surviving to 24.1 years and 25% of patients surviving to 36.5 years of age. For patients born between 1990–99 and 2000–09, the median age was not calculated since more than 50% of the patients were still alive on December 31^st^ 2019 (Fig. 3; population born 1980–2009). The decade-by-decade survival estimations for the DMD population born 1980–2009 did not differ (*p* = 0.92). When analyzing the six healthcare regions of Sweden separately for patients born 1980–2009, the median survival ranged from 26.0 (95% CI 19.1–32.9) to 33.0 (95% CI 27.5–38.5) years of age, with no significant differences except for the Southern region that showed the highest survival. There was no correlation between the age at loss of ambulation and the age at death (Spearman rho = 0.10, *p* = 0.26). There was neither any difference in the age at death between patients losing their ambulation before or after ten years of age (*p* = 0.52).

### Cause of death

**Fig. 4:**
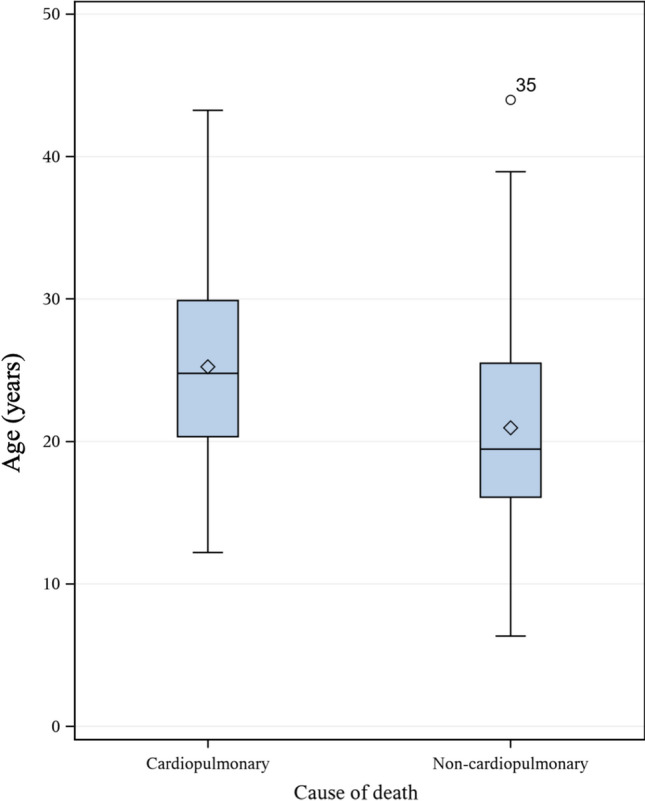
Differences in age between cardiopulmonary and non-cardiopulmonary causes of death in Swedish deceased DMD patients, born 1970–2019. There is a mean difference of 4.28 years (95% CI 1.41–7.33) (*p* = 0.004) between cardiopulmonary causes of death with a mean age of 25.2 (SD 6.5) years compared with non-cardiopulmonary causes of death with a mean age of 21.0 (SD 8.2) years. Line in box represents median age and diamond represents mean age. DMD = Duchenne muscular dystrophy, CI = Confidence Interval, SD = Standard Deviation

**Fig. 5:**
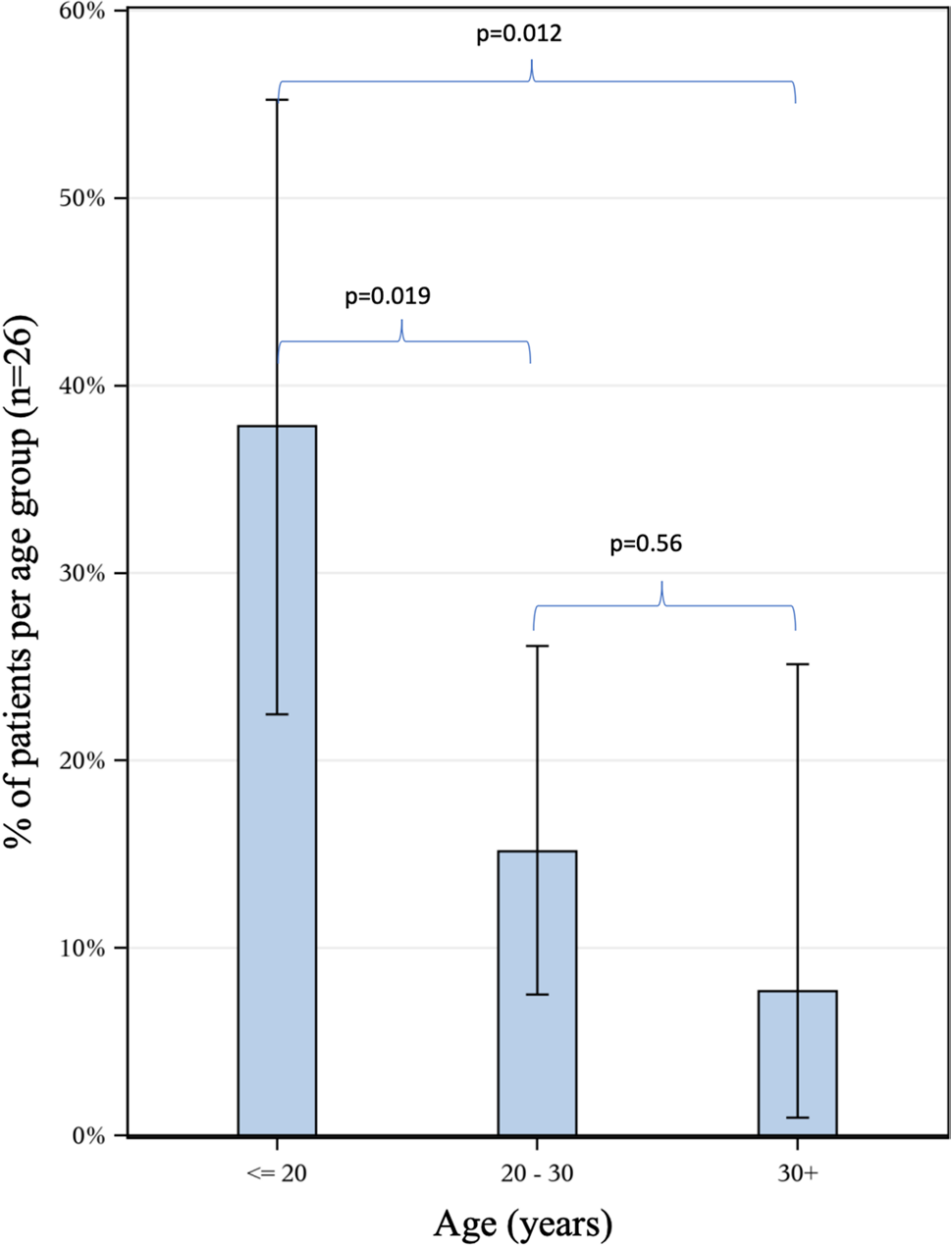
Barchart of the proportion of Swedish deceased DMD patients, born 1970–2019, with non-cardiopulmonary causes of death in each age class; < 20 (n = 14/37; 37.8% (CI 95% 22.5–55.2)), 20–30 (n = 10/66; 15.2% (CI 95% 7.5–26.1)) and 30 + (n = 2/26; 7.7% (CI 95% 1.0–25.1)). DMD = Duchenne muscular dystrophy, n = number, CI = Confidence Interval

The most frequent leading cause of death was cardiac complications (41.9%) (95% CI 33.2–50.9) with a mean age of 26.2 (SD 6.3) years, followed by respiratory failure (38.0%) (95% CI 29.6–46.9) with a mean age of 24.2 (SD 6.6) years. Non-cardiopulmonary complications were the leading causes of death in 20.1% (95% CI 13.6–28.1) of the patients, including: infections (other than pneumonia or myocarditis), stroke, injury-related pulmonary embolism, gastrointestinal complications and unnatural causes (Table 3; population born 1970–2019). Infections as the leading cause of death had an incidence of 1.6 per 1000 person-years, stroke 0.6 per 1000 person-years, injury-related pulmonary embolism 1.3 per 1000 person-years, gastrointestinal complications 1.0 per 1000 person-years, and unnatural causes of death 1.6 per 1000 person-years. Thromboembolic events (including pulmonary embolism and ischemic cerebral infarction) as the leading cause of death had an incidence of 1.6 per 1000 person-years. Death from non-cardiopulmonary complications (mean age 21.0 (SD 8.2) years) occurred in younger age than death from cardiopulmonary complications (mean age 25.2 (SD 6.5) years), with a mean difference of 4.3 years (95% CI 1.4–7.3) (*p* = 0.004), (Fig. 4; population born 1970–2019). Non-cardiopulmonary causes of death were more common for patients under 20 years of age (37.8%) than for patients between 20–30 (15.2%) (*p* = 0.019) and patients over 30 years of age (7.7%) (*p* = 0.012) (Fig. 5; population born 1970–2019). Accidental/non-accidental injury, injury-related pulmonary embolism and stroke as leading causes of death were only present in patients under 25 years of age.

**Table 3:** Causes of death^a^ in Swedish deceased DMD patients, born 1970–2019

		n (%)	Incidence per 1000 person-years
Respiratory			
Acute respiratory failure	Infection	18 (14%)	5.8
	Pneumothorax	2 (1.6%)	0.6
	Secretion stagnation	4 (3.1%)	1.3
	Ventilator error	1 (0.8%)	0.3
	UNS	6 (4.7%)	1.9
Chronic respiratory failure	Chronic respiratory failure	18 (14.0%)	5.8
Cardiac			
Heart failure	Dilated cardiomyopathy	27 (20.7%)	8.8
	Cardiomyopathy UNS	1 (0.8%)	0.3
	Arrythmia	2 (1.6%)	0.6
Myocardial infarction	Myocardial infarction	2 (1.6%)	0.6
Cardiac arrest	Arrythmia	5 (3.9%)	1.6
	Sudden death	10 (7.8%)	3.2
	Related to pneumonia	1 (0.8%)	0.3
	UNS	5 (3.9%)	1.6
Myocarditis	Myocarditis	1 (0.8%)	0.3
Infection			
	Peritonitis	1 (0.8%)	0.3
	Sepsis	1 (0.8%)	0.3
	Pancreatitis	1 (0.8%)	0.3
	Gastroenteritis	1 (0.8%)	0.3
	UNS	1 (0.8%)	0.3
Stroke			
	Ischemic cerebral infarction	1 (0.8%)	0.3
	Subarachnoidal hemorrhage	1 (0.8%)	0.3
Pulmonary embolism			
	Injury-related	4 (3.1%)	1.3
Other			
	Intestinal volvulus	2 (1.6%)	0.6
	Gastrointestinal hemorrhage	1 (0.8%)	0.3
	Acute kidney failure	1 (0.8%)	0.3
	Cancer	1 (0.8%)	0.3
	Post-surgery complications	1 (0.8%)	0.3
	UNS	4 (3.1%)	1.3
Unnatural			
	Accidental/non-accidental	5 (3.9%)	1.6

^a^In 83.7% of the patients the leading causes of death were verified through the medical records. Of the remaining 21 patients (16.3%), the leading cause of death was only based on the Cause of Death Register (non-verified) in 19 patients and was documented as unknown in 2 patients. Generated person-years in deceased group was 3082 years

DMD = Duchenne muscular dystrophy, UNS = unspecified

## Discussion

Our study is one of few nationwide studies to explore the prevalence, life expectancy and leading causes of death in patients with DMD over the past half century. We estimated the birth prevalence of DMD in Sweden to be 19.2 per 100 000 males. Our results confirm previous findings that patients with DMD present an increasing longevity. In our study, the median survival was 29.9 years of age. We found that the age at loss of independent ambulation did not have a significant impact on the overall survival. Cardiac and respiratory failure were the leading causes of death in approximately 80% of our study population. Our study revealed that one in five patients with DMD dies of non-cardiopulmonary causes, and that these causes significantly contribute to higher mortality rates among younger patients with DMD.

We identified four main groups of non-cardiopulmonary causes of death in our study; stroke (2/129 pts); injury-related pulmonary embolism (4/129 pts); gastrointestinal complications (3/129 pts) and unnatural causes of death (5/129 pts). It has been postulated that patients with DMD are at increased risk of thromboembolic events such as cerebral infarctions, deep vein thrombosis and pulmonary embolism, mainly attributed to chronic immobility and underlying cardiac insufficiency [27]. The incidence of thromboembolic events leading to death in our study was estimated to be 1.6 per 1000 person-years (5/129 pts; 3.9%), which is higher than the annual incidence of 57.2 per 100.000 reported in the general population [28]. The incidence of stroke was 0.6 per 1000 person-years (2/129 pts; 1.5%). This is lower than the incidence of ischemic stroke reported in previous studies (0.75–1 per 100 person-years) [29, 30], but notably higher than the incidence found in the Swedish male population under 45 years of age (7.5–11.4 per 100 000) [31]. In addition to the fact that gastrointestinal complications were one of the main non-cardiopulmonary causes of death in our study, gastrointestinal symptoms were reported by Parker et al. as the most commonly reported complaints after orthopedic, respiratory, cardiac and cognitive comorbidities [32]. The incidence of unnatural causes of death in our cohort was 1.6 per 1000 person-years (5/129 pts; 3.9%). Our study is the first to unveil the occurrence of unnatural causes of death and their important effect on mortality in DMD at younger ages.

Cardiac complications were the most frequent leading cause of death in our cohort, with an incidence of 17.2 per 1000 person-years (54/129 pts; 41.9%). Death from cardiac complications occurred later compared to other causes of death, at a mean age of 26 years. There is increasing evidence of a shift in the natural history of DMD to longer life span and death from end-stage heart failure [11]. To our knowledge, our study is the first to present the shift of cardiac-related mortality towards older ages compared with mortality due to respiratory failure [10, 18]. Improvements in respiratory care and assisted ventilation over the years, as well as the introduction of cardio-protection are considered major contributors to this shift [19, 20, 33]. Other comorbidities that come with aging, such as hypertension and diabetes type 2, have been relatively uncommon in DMD. Still, physicians treating older patients with DMD will be challenged with a new multi-comorbid panorama as part of the evolving natural history of the disease.

Pneumonia-induced acute respiratory failure was encountered as the leading cause of death in 14% of the patients (incidence 5.8 per 1000 person-years). Chronic bacterial colonization of the upper airways is common among patients with neuromuscular disease, causing airway inflammation and recurrent respiratory tract infections with a significant effect on DMD morbidity and mortality. Adherence with respiratory care guidelines, especially in the setting of acute respiratory illness, although crucial, is lacking [34]. New therapeutic modalities, such as inhaled antibiotics, are emerging in the respiratory management of neuromuscular diseases, potentially changing the respiratory outcomes and related mortality rates.

Our study reports a median life span of 30 years of age, wich is in accordance with other studies of mixed DMD population with different treatment regimes [35]. The increased life span for patients with DMD over time reflects not only the evolution of standards of care in DMD worldwide [6], but also an overall improvement of patient management on a national level. The effect of wheelchair confinement on life expectancy has been debated. We found that age at loss of ambulation does not correlate to the age of death in DMD, confirming similar observations [32]. Age at loss of ambulation has been considered as an indicator of the disease severity in DMD, and may contribute to other comorbidities, such as progressive scoliosis and pulmonary decline, but has no impact on survival according to our study. The fact that there were no major differences in patient longevity between regions is believed to be the result of a continuous work towards harmonization of care across healthcare providers and regions, as well as the implementation of a follow-up multidisciplinary program in specialized clinics for patients with neuromuscular diseases in Sweden. Harmonization of patient care and access to specialized teams of healthcare professionals is of vital essence not only for DMD but also for all rare diseases, especially in the era of disease-modifying novel therapies that change the natural history of the disease.

A strength of this study is the thorough, multisource patient identification and data collection, which ensured a high calculated birth prevalence of 19.2 and 20.5 per 100,000 male births for patients born 1980–89 and 1990–99. This is, however, a retrospective, nationwide cohort study of patients born over the past five decades, generating some limitations worth to be mentioned. First, finding all patients with DMD diagnosis born since 1970 was challenging, especially for patients born during the first study decade. Furthermore, identifying a single leading cause of death in DMD patients was difficult in some cases, as there could be multiple conditions contributing to death, such as a combination of chronic cardiac and pulmonary failure. Moreover, some patients died at home as a result of their chronic condition, without further medical examination or postmortem investigation. To minimize unidentified cases and augment data validity, we have used multiple sources for patient identification and data verification, as well as a standardized methodological approach to determine the leading cause of death, and succeeding to verify the cause of death with medical reports in 83.7% of the cases; still, few patients and some medical information may have been missed. Patients born before 1980 were not included in the survival analysis. The reason for this is the low calculated birth prevalence for patients born during this decade. Moreover, many of the patients born 1970–79 were identified through Swedevox, suggesting that the majority of these patients had reached an age requiring non-invasive ventilatory support and survival analysis was therefore at risk of being skewed towards older ages.

In conclusion, improvements in the care of patients with DMD have also affected survival and causes of death in a national perspective. The fact that one in five patients with DMD dies from non-cardiopulmonary causes, and that these causes contribute significantly to higher mortality among younger patients with DMD, suggests that deeper research is needed on risk-factor identification and prevention. The changing epidemiology of DMD and the new disease panorama of the growing adult DMD population requires reorientation of health care, both in preventive measures at young ages and tackling new comorbidities in older patients with DMD.

## Data Availability

Case report form is available upon request.
